# A comparative histological study of the osteoderms in the lizards *Heloderma suspectum* (Squamata: Helodermatidae) and *Varanus komodoensis* (Squamata: Varanidae)

**DOI:** 10.1111/joa.13156

**Published:** 2020-01-27

**Authors:** Alexander Kirby, Matthew Vickaryous, Alan Boyde, Alessandro Olivo, Mehran Moazen, Sergio Bertazzo, Susan Evans

**Affiliations:** ^1^ Department of Medical Physics and Biomedical Engineering University College London London UK; ^2^ Department of Biomedical Sciences University of Guelph Guelph ON Canada; ^3^ Dental Physical Sciences, Barts and The London School of Medicine and Dentistry Queen Mary University of London London UK; ^4^ Department of Mechanical Engineering University College London London UK; ^5^ Department of Cell and Developmental Biology University College London London UK

**Keywords:** Gila monster, histology, Komodo dragon, lizard, osteodermine, osteoderms, polarised light

## Abstract

We describe the histological appearance of the osteoderms (ODs) of *Heloderma suspectum* and *Varanus komodoensis* using multiple staining and microscopy techniques to yield information about their morphology and development. Histological analysis showed that the ODs of *H. suspectum* are composed of three main tissue types, a superficial layer, herein identified as osteodermine, capping a base composed of Sharpey‐fibre bone and lamellar bone rich in secondary osteons (Haversian bone tissue). In contrast, ODs in *V. komodoensis* are composed of a core of woven bone surrounded by parallel‐fibred bone without a capping tissue. Thus, in these two species, ODs differ both in terms of their structural composition and in details of their skeletogenesis. The histology of the mineralised tissues observed in these two reptile taxa provides insights into the mechanism of formation of lizard ODs and presents a direct comparison of the histological properties between the ODs of the two species. These data allow greater understanding of the comparative histological appearance of the dermal bones of lizards and highlight their structural diversity.

## Graphical abstract

The histological structure of the osteoderms (ODs) of two lizard species are described and compared. Results show that, although the two species are closely related, the histology of the ODs are notably different and may follow different developmental paths.

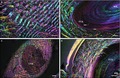

## 1 INTRODUCTION

Osteoderms (ODs), a term which literally means 'bone in the skin', are hard tissue organs embedded into the dermis of vertebrates (Moss, 1972), forming part of the dermal (integumentary) skeleton of tetrapods (Vickaryous and Sire, 2009). They are often referred to as 'ossicles', 'bony plates' or 'dermal armour', among other synonyms. The histological organisation of skeletally mature ODs provides information about their mode of development (Moss, 1972; de Buffrénil et al. 2010; de Buffrénil et al. 2011), evolutionary origin, and homology across species (de Buffrénil et al. 2010). Previous work has shown that OD development is not homogenous across vertebrates (Vickaryous and Sire, 2009); the mode of development of some ODs in mammals, e.g. in *Dasypus novemcinctus* (Linnaeus, 1758)*,* the nine‐banded armadillo, is comparable with intramembranously derived elements of the human skull (Vickaryous and Sire, 2009). However, in reptiles, including dinosaurs (e.g. Horner et al. 2016), alligators (Vickaryous and Hall, 2008) and lizards (Zylberberg and Castanet, 1985; Levrat‐Calviac and Zylberberg, 1986; Vickaryous et al. 2015), ODs have been postulated to arise via spontaneous mineralisation that forms within pre‐existing dermal collagen arrangements, much like tendon ossification (Hall, 2015).

Among living tetrapods, lizards (exclusive of snakes) include the largest number of OD‐bearing taxa. In this group, ODs are considered to represent a primitive trait of the basal tetrapods, lost in some later lineages (Levrat‐Calviac and Zylberberg, 1986), and related to the latent ability of the dermis to generate a mineralised tissue (Main et al. 2005). ODs' expression within a clade is often variable, even within a single genus (Campbell, 1982). Lizard ODs have a heterogeneous tissue composition and have been described as containing woven bone (Zylberberg and Castanet, 1985), parallel‐fibred bone (Vickaryous and Hall, 2008), lamellar bone (LB; Zylberberg and Castanet, 1985; de Buffrénil et al. 2010) and Sharpey‐fibre bone (SFB; Vickaryous and Hall, 2008; Vickaryous et al. 2015). This diversity in OD composition, morphology, arrangement and distribution makes lizards an ideal group in which to examine the differences in OD histology.

A recently discovered highly mineralised OD component, osteodermine, was first named in a fossil squamate (Glyptosaurinae) from the late Cretaceous (de Buffrénil et al. 2011). Since then, this material has been observed on the superficial surface of extant *Tarentola annularis* (Geoffroy Saint‐Hilaire, 1827; Vickaryous et al. 2015) and *Tarentola mauritanica* ODs (Linnaeus, 1758; Levrat‐Calviac and Zylberberg, 1986)*.* Relatively little has been done, however, to characterise osteodermine histologically, nor has there been much research as to the latent ability of squamates to mineralise dermal components (Haines and Mohuiddin, 1968). Indeed, the most recent review of tetrapod ODs (Vickaryous and Sire, 2009) highlighted the gaps in our current knowledge of the skeletogenesis and subsequent material composition of squamate ODs and called for further investigation into these. A recent study compared cell‐mediated mineralisation of ODs to that of heterotopic ossification in humans (Dubansky and Dubansky, 2018), emphasising the importance of understanding the ontogeny of ODs in the context of creating models for human disease.

*Varanus komodoensis* (Ouwens, 1912; Komodo dragon) and *Heloderma suspectum* (Cope, 1869; Gila monster) are two relatively closely related (Varanidae and Helodermatidae, respectively) anguimorph squamates known to possess ODs. These ODs are distinctive among lizards, in that they are separated from one another by soft tissue, rather than forming a dense protective sheet of overlapping ‘tiles’ as in most scincoid and anguid lizards. Nonetheless, there are differences in the gross morphology and arrangement of the ODs in *H. suspectum* and *V. komodoensis*. In the former, the ODs form a stud‐like body covering of isolated, rounded mounds, and in the latter, slender, vermiform cylinders, as well as rosette and dendritic‐shaped ODs mesh into an arrangement that is more like ‘chain mail’ (Maisano et al. 2019). This may reflect the contrasting activity patterns of the two species; the Komodo dragon is an active predator of large mammals, whereas the Gila monster is sluggish and spends most of its time underground.

The ODs of *H. suspectum* have been described as being composed of a basal layer of bone, but having a capping tissue of unknown composition and origin (Moss, 1972; Vickaryous and Sire, 2009). Our objectives were, firstly, to provide a detailed histological characterisation of ODs from the anguimorphs *H. suspectum *and* V. komodoensis* and, secondly, to characterise the unknown capping tissue in *H. suspectum* to test if it is osteodermine and to determine whether it is present in the ODs of *V. komodoensis*, the histology of which is currently undescribed.

## 2 METHODOLOGY

### 2.1 Skin samples

For the purposes of the present study, samples of skin measuring ~10 cm^2^ were dissected from the post‐cranial dorsum of *H. suspectum* and *V. komodoensis* and frozen at −20°C. The lizard samples were provided by the Zoological Society of London, London Zoo Pathology Department (to author Evans).

### 2.2 Histology

Dissected skin samples were defrosted and fixed in 10% neutral buffered formalin for 24 h, then placed in decalcification solution (1.9% glutaraldehyde, 0.15 M EDTA, in 0.06 M sodium cacodylate buffer, adjusted to 7.4 pH) at 4°C, changing to fresh solution every 7 days, for 4 weeks. Further decalcification was performed prior to embedding using a Sakura TDE™30 Electrolysis Decalcifier System (Item Code 1427) and Sakura TDE™30 Decalcifier Solution (Item Code 1428) on default settings. Samples were embedded in paraffin wax and then sectioned either coronally or parasagittally, at 5‐μm thickness, with an HM Microm 355S automatic rotary microtome (Thermo Fisher Scientific). These sections were stained with haematoxylin and eosin (H&E; Kiernan et al. 2010), Alcian blue (Klymkowsky and Hanken, 1991), Masson's trichrome (Calvi et al. 2012) or Elastic Verhoeff van Gieson (EVG; Puchtler and Waldrop, 1979) (Table S1). The slides were scanned using a Leica SCN400 scanner to create a digital image.

### 2.3 Cross‐polarised light microscopy

For cross‐polarised light microscopy, samples were prepared as above for histology, but prior to staining procedures, the dewaxed, unstained sections collected on glass slides were stained with 0.5% aqueous toluidine blue on a heated stage for 15 s and the result observed through crossed polars on a Zeiss LSM 510 Meta Laser scanning microscope.

### 2.4 Multi‐rotation polarised light microscopy

Multi‐rotation polarised light microscopy was achieved by rotating crossed‐polarising filters around a stationary sample, a new approach to increase the information content in polarised light microscopy of all tissues, introduced by Boyde et al. (2019). In the present study, the overwhelming polarised light microscopy signal was attributable to the positive form birefringence of collagen. By sampling at close rotation intervals of 15°, the signal due to collagen fibre orientation was constant (to within 96.7%: cos 15° = 0.9659), irrespective of its axis in the plane of section. For the present study, we used automated rotation of the polarising and analysing filters at six 15° intervals, with linearly polarised light images recorded at each orientation. For reference, the objectives used were 4/0.13, 10/0.30, 20/0.50 and 40/0.75. The images were merged using ImageJ in the colour circular sequence red (R), yellow (Y), green (G), cyan (C), blue (B), magenta (M; importantly, ensuring that the intensities generated by the intermediate Y, C, M colours matched those of the three primary colours, RGB). Colour in the composite image shows the orientation within the section plane, with four repeat cycles in 360°. Brightness was proportional to the cosine of the strike angle with respect to section plane, being brightest in plane, and black when perpendicular to that plane, i.e. parallel to the optic axis. For the present study, we used H&E‐ and Masson's trichrome‐stained sections. Prior staining makes some difference to the light absorption, but this did not contribute to the output colour in the combined images.

### 2.5 X‐ray plate imaging

Dissected samples were placed onto the plate detector of a Nomad Pro 2 X‐ray system and exposed to X‐rays from 30 cm of distance with a tube voltage of 60 kV and a minimum current of 2.5 mA, for a duration of 0.2 s. The digital file was scaled using ImageJ with a control image of a radiopaque ruler.

## 3 RESULTS

**Figure 1:**
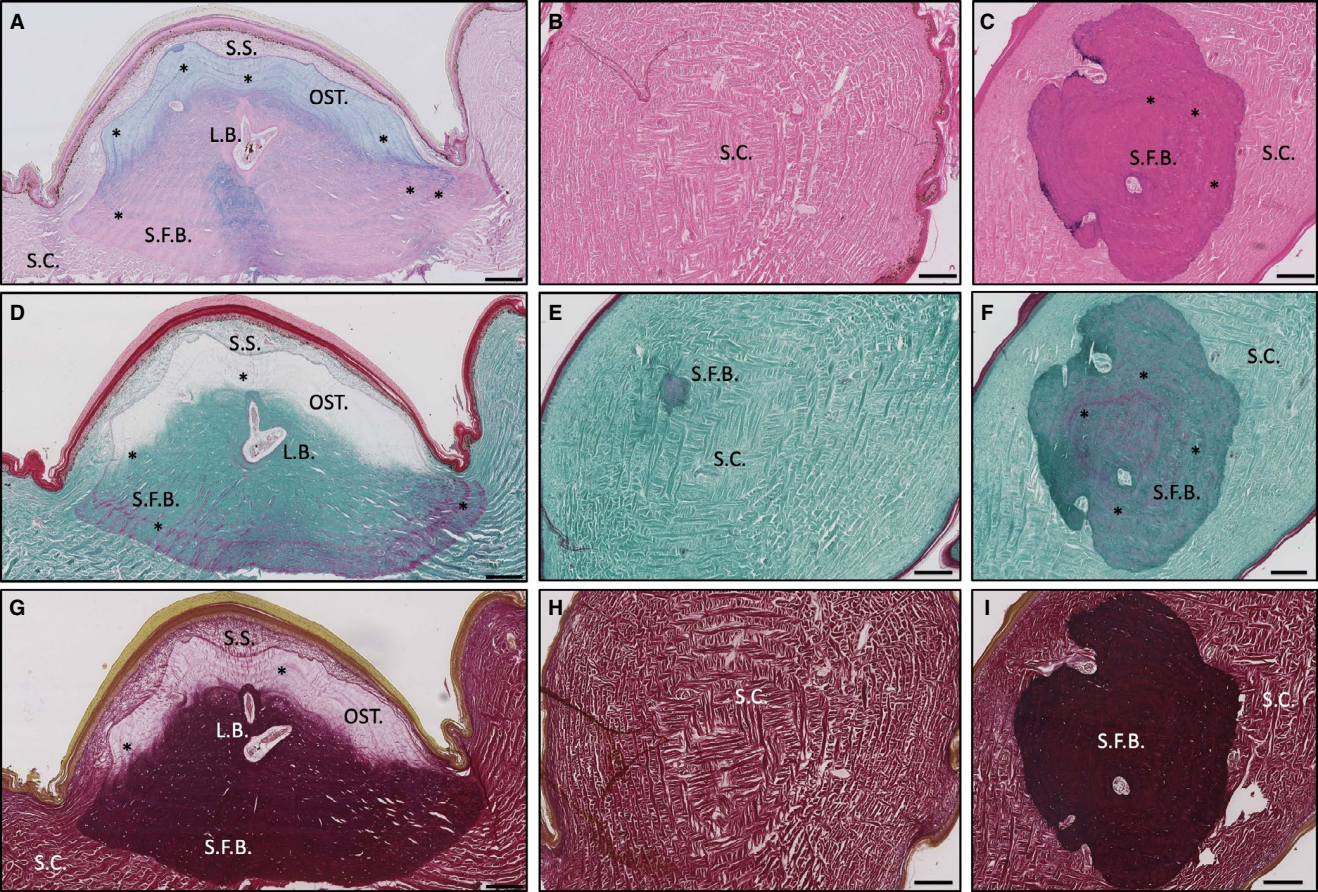
Histological overview of *Heloderma suspectum* osteoderm in (A,D,G) parasagittal and (C,F, I) coronal sections stained with (A) Alcian blue, (B,C) haematoxylin and eosin, (D–F) Masson's trichrome and (G–I) Elastic Verhoeff van Gieson. Also (B,E,H) coronal sections of unmineralised dermis for comparison. L.B., lamellar bone; OST., osteodermine; S.C., stratum compactum; S.F.B., Sharpey‐fibre bone; S.S., stratum superficiale. Asterisks indicate growth lines. Scale bars: all 200 μm.

Selected histological stains (Table S1) were implemented to aid the visualisation of the structural morphology of the ODs from both species. When viewed in the dorsoventral plane using X‐ray plate imaging, the ODs of *H. suspectum* appeared as non‐overlapping, circular shapes, roughly 2–4 mm in diameter, regularly tessellated with hexagonal symmetry and displaying a rough, ornamented superficial surface (Fig. S1A). In parasagittal section (Fig. 1A), the ODs of *H. suspectum* appeared lozenge‐shaped with a vermiculate superficial surface, and a smooth deep surface, situated at the interface between the deep dermis [stratum compactum (SC)] and the superficial dermis [stratum superficiale (SS)] (Fig. 1B). In places, the apical surface of the OD resided in close contact (<20 μm) with the stratum germinativum of the epidermis (Fig. 1A), while the basal surfaces were located at a greater distance from the epidermis (Fig. 1C). Each OD was overlain by a single keratinised 'scale' or 'scute' (not to be confused with mineralised scales of fish), which stained bright red with Masson's trichrome staining (Fig. 1D, outermost layer).

In *H. suspectum,* each OD was composed of three distinct mineralised tissues: (a) osteodermine; (b) SFB; and (c) LB. The base of each OD was composed of an ossified tissue that incorporates numerous thick collagen bundles passing from the adjacent SC. Based on comparisons of similar ossified tissues, rich in large collagenous fibres (e.g. Witten and Hall, 2002; Vickaryous et al. 2015), we interpret this as SFB. Weak staining with Alcian blue (Fig. 1A) indicated the presence of acid mucosubstances within the SFB, although no evidence of cartilage was observed. Masson's trichrome staining (Fig. 1D–F) revealed the characteristic presence of numerous large bundles of collagen fibres within the SFB, some of which were under tension (i.e. stained red; as shown under experimental tension by Flint and Merrilees, 1976), while EVG staining (Fig. 1G–I) showed that few elastin fibres were present. The SFB was cellular, as evidenced by various osteocytes, and showed evidence of repeating darkly stained lines, indicative of growth and/or mineralisation fronts (Fig. 1A,D, asterisks).

Invested within the SFB were deposits of concentrically organised LB around endosteal surfaces of the medullary cavities and neurovascular canals, creating Haversian systems or secondary osteons (LB; Fig. 1). Unlike SFB, LB failed to stain with Alcian blue (Fig. 1A), but did show evidence of collagen bundles in tension (with Masson's trichrome; Fig. 1C) and the presence of elastin fibres in the lumen of the canal (EVG staining; Fig. 1G).

Capping each *H. suspectum* OD residing in the non‐mineralised SS was an unusual tissue, herein identified as osteodermine (Fig. 1A,D,G). Matching previous reports (de Buffrénil et al. 2011; Vickaryous et al. 2015), *H. suspectum* osteodermine is collagen‐poor, essentially acellular, and demonstrates evidence of periodic growth. *H. suspectum* osteodermine stained weakly for Alcian blue (Fig. 1A), indicating the presence of acid mucosubstances, lacked intrinsic collagen (as revealed by Masson's trichrome; Fig. 1D) and elastin (using EVG; Fig. 1G), and showed evidence of periodic growth (Fig. 1, asterisks).

**Figure 2:**
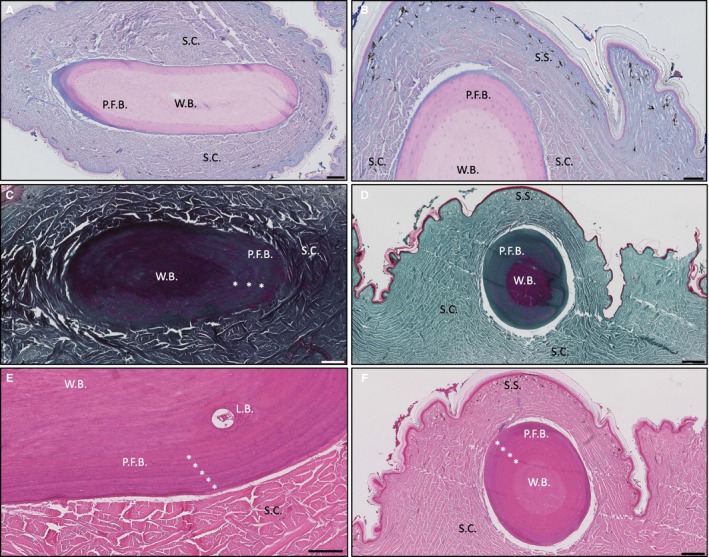
Histological overview of *Varanus komodoensis* osteoderm stained with Alcian blue in (A) parasagittal and (B) coronal section, stained with Masson's trichrome in (C) parasagittal and (D) coronal section; and stained with haematoxylin and eosin in (E) parasagittal section and (F) coronal section. L.B., lamellar bone, P.F.B., parallel‐fibred bone, S.C., stratum compactum; S.S., stratum superficiale; W.B., woven bone. Asterisks indicate growth lines. Scale bars: (A) = 200 μm, (B,C) = 100 μm, (D) = 200 μm, (E) = 100 μm and (F) = 200 μm.

In contrast to the robust, bead‐shaped ODs of *H. suspectum*, those of *V. komodoensis* resembled smooth, slightly bent cylinders, ~2–4 mm in length and partially overlapping one another (Fig. S1B). Accordingly, the morphology of these elements has been described as vermiform (e.g. Erickson et al. 2003). In addition to gross morphology, ODs in *V. komodoensis* also differ from those of *H. suspectum* at the level of serial histology. For example, in *V. komodoensis*, ODs were found invested within the deep dermis (SC; Fig. 2A), while those of *H. suspectum* were located at the interface between the superficial (SS) and deep dermal compartments. In addition, *V. komodoensis* ODs lacked SFB and osteodermine. Instead, they were primarily composed of: (a) woven bone; (b) LB; and (c) parallel‐fibred bone. In section, *V. komodoensis* ODs had a conspicuous concentric arrangement of outer rings and an inner core (Fig. 2). The inner core region was composed of woven bone, exhibiting a densely packed arrangement of interlacing, randomly arranged mineralised collagen fibres. The woven bone core was negative for acid mucosubstances (Fig. 2A,B) and showed evidence of collagen fibres in tension (Fig. 2C,D). The outer rings were composed of parallel‐fibred bone, with closely packed and regularly arranged collagen fibres. The periphery of each OD is surrounded by a thin layer of Alcian blue‐positive osteoid (unmineralised bone; Fig. 2A,B). We also observed evidence of remodelling, with deposits of LB contributing the formation of an osteon (Fig. 2E), as well as concentric lines of growth (Fig. 2F, asterisks).

**Figure 3:**
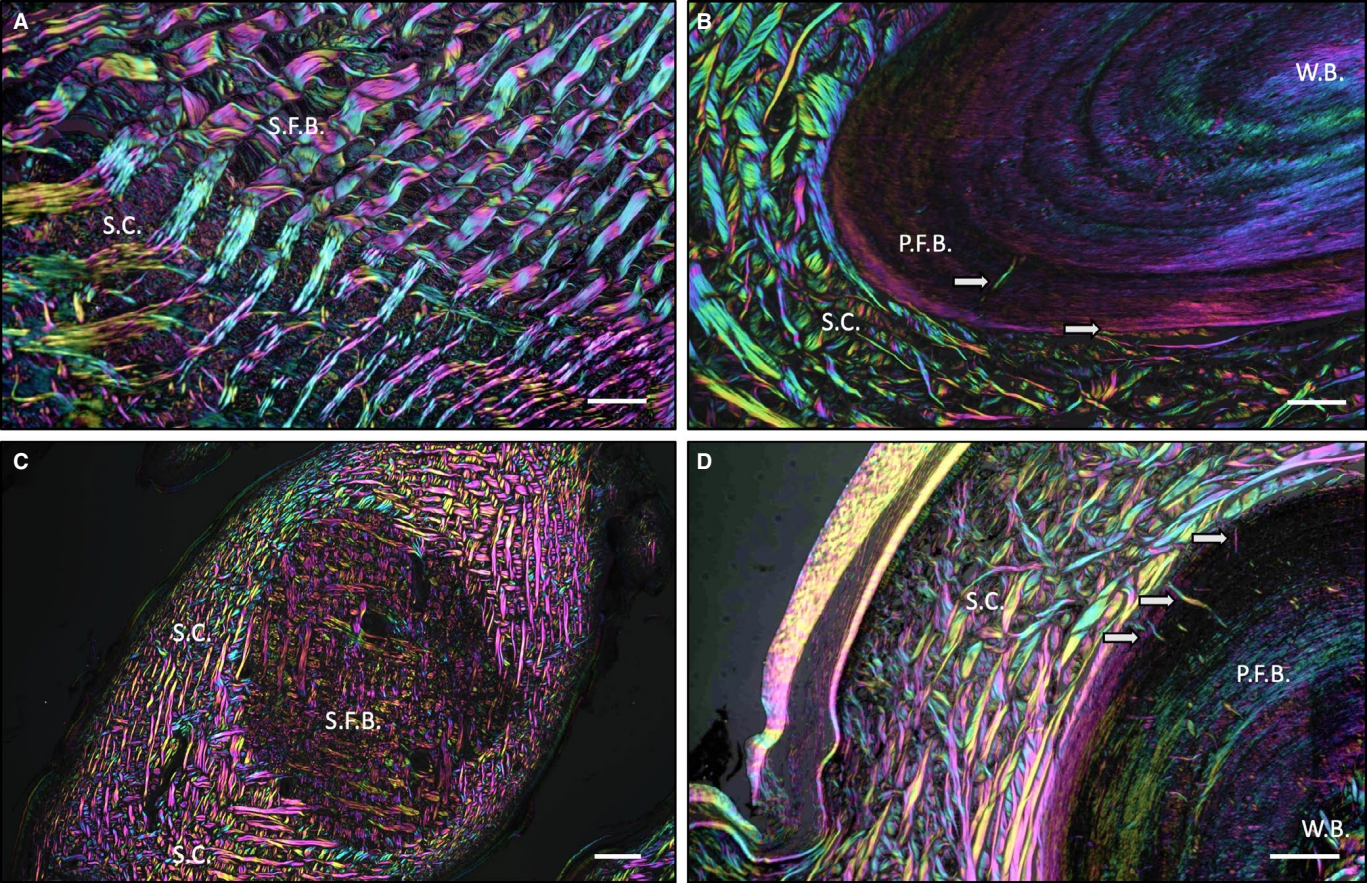
(A) Parasagittal section of *Heloderma suspectum* osteoderm (OD), (B) parasagittal section of *Varanus komodoensis* OD, (C) coronal section of *H. suspectum* OD, and (D) coronal section of *V. komodoensis* OD. (A–D) All stained with haematoxylin and eosin and visualised with multi‐rotation polarised light microscopy. P.F.B., parallel‐fibred bone; S.C., stratum compactum; S.F.B., Sharpey‐fibre bone; W.B., woven bone. White arrows indicate Sharpey's fibres. Scale bars: (A) = 50 μm, (B) = 50 μm, (C) = 250 μm and (D) = 50 μm.

To further investigate the relationship between ODs and the dermis, we used polarised light microscopy (Fig. 3). In *H. suspectum* ODs, the boundary between the mineralised (SFB) and the non‐mineralised SC was almost indistinguishable, with regularly interspaced bundles of collagen fibres of the dermis passing uninterrupted into the mineralised OD (Fig. 3A). In contrast, for *V. komodoensis* ODs, there was a sharp boundary between bone and dermis, with only occasional Sharpey's fibres anchoring the skeletal element into the surrounding skin (Fig. 3B, white arrows). The same circumstances were observed in coronal sections of both species (Fig. 3C,D).

**Figure 4:**
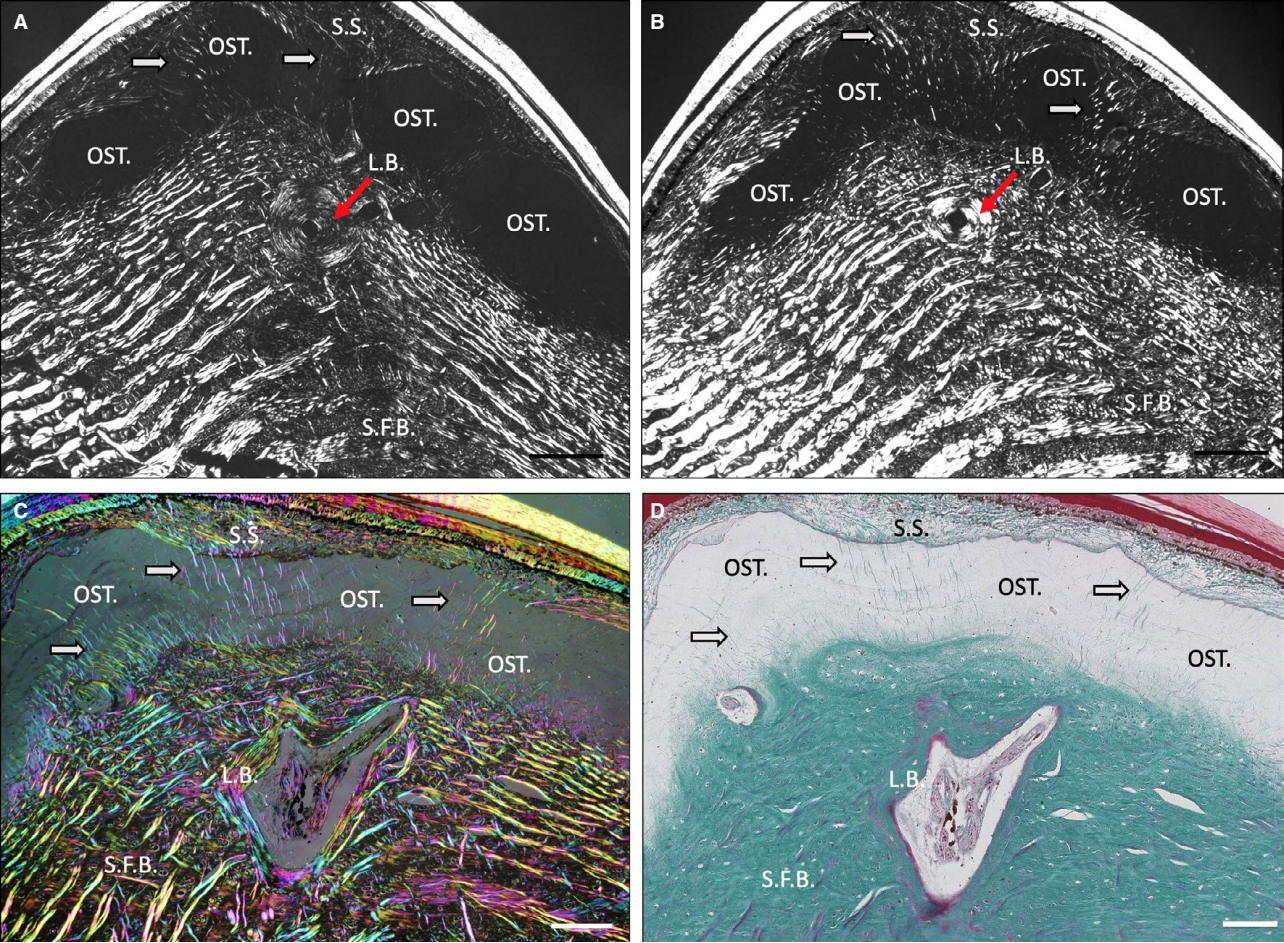
(A,B) Parasagittal sections of *Heloderma suspectum* osteoderm (OD) stained with toluidine blue, visualised with cross‐polarised light microscopy. (C) *H. suspectum* OD, parasagittal section stained with Masson's trichrome, visualised with multi‐rotation polarised light microscopy and (D) same section as (C), visualised with light microscopy. L.B., lamellar bone; OST., osteodermine; S.C., stratum compactum; S.F.B., Sharpey‐fibre bone; S.S., stratum superficiale. Red arrows indicate L.B. White arrows indicate Sharpey's fibres. Scale bars: All 100 μm.

In *H. suspectum,* osteodermine failed to stain with toluidine blue and displayed monorefringence when viewed using polarised light, with evidence of concentrically arranged lines of arrested growth (osteodermine; Fig. 4A–D). As revealed by staining with Masson's trichrome, intrinsic collagen fibres appeared to be virtually absent from the osteodermine matrix, whereas penetrating Sharpey's fibres were observed passing radially through the otherwise vitreous matrix (Fig. 4C,D, white arrows). A 'Maltese cross', indicative of a secondary osteon following bone remodelling (Stump, 1925), was observed in the LB region across multiple sections as a cross‐shaped, dark formation in transmitted light (LB; Fig. 4A,4, red arrows). Occasionally, we were able to identify vascular channels that were not encased by this LB and therefore did not produce a Maltese cross (Fig. 4A,B). We were not able to identify a Maltese cross in any *V. komodoensis* section.

## 4 DISCUSSION

The primary objective of this study was to provide a detailed histological characterisation of ODs from the anguimorphs *H. suspectum* and *V. komodoensis*. The second objective was to characterise the histological appearance of osteodermine and to confirm its presence in *H. suspectum* ODs.

With regard to the first objective, histological results demonstrated several important differences in the staining and structure of the mineralised materials present in the ODs of *H. suspectum* and *V. komodoensis*. Whereas ODs of both species are present within the dermis and are primarily composed of bone, their relative placement within the dermis and the structural organisation of the ossified matrix differs notably between the two species. In addition, *H. suspectum* develops a conspicuous capping tissue (herein identified as osteodermine), whereas *V. komodoensis* does not. Taken together, these data suggest that disparity in OD structure reflects fundamental differences in the functional role and evolutionary history of these elements. As explained in the introduction, Gila monsters are understood to be lethargic hunters, whereas Komodo dragons are active hunters. Therefore, it seems likely that differences in OD structural composition reflect the different ecological environments of the dermis within the distinct predatorial niches that these two species occupy. Accordingly, future studies that attempt to elucidate a clear correlation between ecological environment and the histology of lizard ODs would be welcomed.

Whereas the dermis represents the structural milieu of OD formation, there are differences across species in the relative position of the skeletally mature elements. As observed in *Varanus salvator* (Erickson et al. 2003), the ODs of *V. komodoensis* were situated comparatively deep within the SC of the dermis, whereas those of *H. suspectum* were located at or near the interface between the SS and SC*.* A similar position has also been described for the OD (and osteodermine)‐bearing geckos *T. mauritanica* and *T. annularis* (Vickaryous et al. 2015). This raises the possibility that the formation of osteodermine may require proximity to the SS (and possibly even the adjacent epidermis; de Buffrénil et al. 2011). For now, however, the developmental origins of osteodermine remain uncertain.

Mineralised collagen was found to be present throughout the ODs of *V. komodoensis* but, whereas it stained green in Masson's trichrome in the outer region, the inner region stained red, indicating that the collagen is under tension within the core (Fig. 2D). *H. suspectum *ODs showed the reverse condition, where more green staining occurred within the base and core of OD compared to more red staining in the periphery and the complete lack of stain in the superficial osteodermine regions (Fig. 1D). These differences in the distribution and tension of collagen within the ODs of the two species may affect the mechanical properties of the ODs under load and may be related to differences in the ecology of the two species.

Both *H. suspectum* and *V. komodoensis* demonstrate a comparable orthogonal arrangement of large collagen bundles within the dermis (SC and SS; Figs 1 and 2), and ODs from both species demonstrate a conspicuous basophilic line demarcating the limit of mineralisation, similar to that reported for *Heloderma horridum* (Moss, 1969). Both species of *Heloderma*, but not *Varanus*, preserve the same herringbone organisation of collagen within the matrix of the tissue we identify as SFB (Fig. 1C,1,I; Moss, 1969). SFB in *Heloderma* exhibited multiple lacunae, and occasionally horizontal, repeating darker lines of growth towards the basal layers of the OD (Fig. 1A,1, asterisks), indicating mineralisation may occur radially from an initial central point, as reported in alligator ODs (Vickaryous and Hall, 2008). Both squamate and crocodilian ODs (de Buffrénil et al. 2010, 2015) have been hypothesised to originate via the formation of an initial nucleus of mineralisation, the developmental origins of which are currently uncertain, followed by remodelling to yield LB of osteoblastic origin. This could also be the path of development followed in helodermatid ODs, but further developmental studies are required to determine whether this is the case.

Unlike ODs in *Heloderma*, those of *V. komodoensis* did not demonstrate SFB, suggesting that the original arrangement of collagen fibres from the dermis of varanids were replaced and/or remodelled by newly laid fibres of parallel‐fibred bone (Fig. 2E)*.* Indeed, the mineralised collagen fibres of parallel‐fibred bone were seen to be arranged in rings around a central core, with disruption to this lamellar orientation in the inner core where the collagen fibres became more woven and randomly orientated (Fig. 2D,2). When the parallel‐fibred bone was compared to the mineralised mesh of orthogonal fibres observed in the SFB region of *H. suspectum* (Fig. 1C,1,I), it was evident that *V. komodoensis* ODs exhibit different mineralised collagen structures (Fig. 2D,2). LB was identified surrounding internal vasculature in *V. komodoensis* ODs (Fig. 2E), but the ODs of *V. komodoensis* were rarely seen to be vascularised in comparison to *H. suspectum* ODs.

The results presented here provide evidence that the collagen structure of the dermis is not mirrored in the mineralised collagen observed within *V. komodoensis* ODs, as is the case in *H. suspectum* ODs*.* We observed a similarity between the radial concentric lines of arrested growth in the ODs of *V. komodoensis* (Fig. 2, asterisks) and those of *H. suspectum* ODs (Fig. 1, asterisks). These also resemble OD growth lines previously documented and used to estimate the age of specimens of *V. salvator* (Erickson et al. 2003). We showed that growth lines were also visible in osteodermine of *H. suspectum* ODs, as in *T. mauritanica* ODs*,* where previous authors regarded them as an artefact of spheritic (globular) mineralisation (Levrat‐Calviac and Zylberberg, 1986).

We observed LB in structures that resemble osteons and that display a Maltese cross formation when observed with polarised light microscopy and a 'scalloped border' (Vickaryous and Hall, 2008) in histological staining. This suggests that remodelling of the *H. suspectum* OD may occur. The presence of osteons as Haversian structures, in direct association and contact with SFB, suggests multiple mechanisms of formation in the ontogenesis of *H. suspectum* ODs, an initial mineralisation (not yet understood), then remodelling to form secondary Haversian structures (de Buffrénil et al. 2011), followed by the deposition of the osteodermine cap (Vickaryous et al. 2015).

With regard to the second objective, assessing the histology of osteodermine and its distribution in *H. suspectum*, based on the below criteria, we identified the capping material on the ODs of *H. suspectum* as osteodermine. Histologically, osteodermine is characterised as a vitreous, highly mineralised and cell‐poor tissue that lacks intrinsic collagen (de Buffrénil et al. 2011; Vickaryous et al. 2015). Osteodermine demonstrates weak staining for Alcian blue, indicating the presence of acid mucosubstances, but is not metachromatic with Toluidine blue; in addition, *H. suspectum* osteodermine is not birefringent under polarised light. At of the time of publication, osteodermine has now been identified in species of two distantly related genera: *H. suspectum* (the present study), *H. horridum* (Moss, 1969), *T. mauritanica* and *T. annularis* (Vickaryous et al. 2015), suggesting that the taxonomic distribution of this tissue remains to be recorded. Considering the homology of osteodermine across species, we were able to identify all the features previously used to define osteodermine in the sampled skin *H. suspectum*, and did not identify any additional, novel features in *H. suspectum* osteodermine. The ODs of *H. suspectum* are relatively large and are shown here as having a very thick cap of osteodermine, thicker than in previously studied species (Vickaryous et al. 2015). *H. suspectum* is therefore a prime candidate species for any further research into this material.

In conclusion, the basal regions of *H. suspectum* ODs were found to be composed of SFB containing mineralised dermal collagen fibres, with LB sometimes found surrounding internal vasculature. Osteodermine was identified as the previously unnamed capping material (Moss, 1969). The ODs of *V. komodoensis* were found to be primarily composed of outer rings of parallel‐fibred bone, with an inner core of woven bone, without evidence of osteodermine or SFB. LB surrounding internal vasculature was observed once in *V. komodoensis* ODs, but they are rarely vascularised in comparison to *H. suspectum.* The results of the present study underscore the histological variability observed in ODs within lizards, and suggest that the formation of ODs in *Varanus spp.* and *Heloderma spp.* may follow different developmental paths, despite being relatively closely related.

## CONFLICTS OF INTEREST

None declared.

## Data Availability

All relevant data are presented in the results section and figures.
