# Supplementary material for: A comparative histological study of the osteoderms in the lizards Heloderma suspectum (Squamata: Helodermatidae) and Varanus komodoensis (Squamata: Varanidae)
Source: J Anat. 2020 Jan 27;236(6):1035–43. doi: 10.1111/joa.13156 (PMC7219622; doi:10.1111/joa.13156)
Supplement: Supplementary file 1 [file JOA-236-1035-s001.docx]

**Supplementary Material:**

**Histochemical** **stain**

H&E (Haematoxylin and eosin)

Masson’s Trichrome

E.V.G./V.V.G. (Elastic/Verhoeff Van Gieson)

Alcian Blue

Toluidine Blue

**Utility**

Shows general nuclear and cytoplasmic morphology.

Shows collagen and keratin

Shows elastic fibres

Shows acid mucosubstances/ carboxylated glycosaminoglycans and acid mucins

Standard Light: Shows general morphology/ mineralisation of bone matrix

Polarised light: Arrangement, orientation of collagen

**Colour results**

Nuclei: Blue

Cytoplasm, collagen: Pink

Bone, osteoid: Pink-Purple

Collagen: Green/Blue

Nuclei: Black

Muscle, cytoplasm, keratin: Red

Collagen: Red

Elastic fibres: Black

Acid mucosubstances: Blue

Nuclei: Red

Cytoplasm: Pink

Acidic tissue compounds (incl. DNA, RNA): Blue

Collagen: Birefringent blue/orange

**Supplementary Table 1:** A list of included histological stains, a description of their general characteristics and colour interpretation of the results.


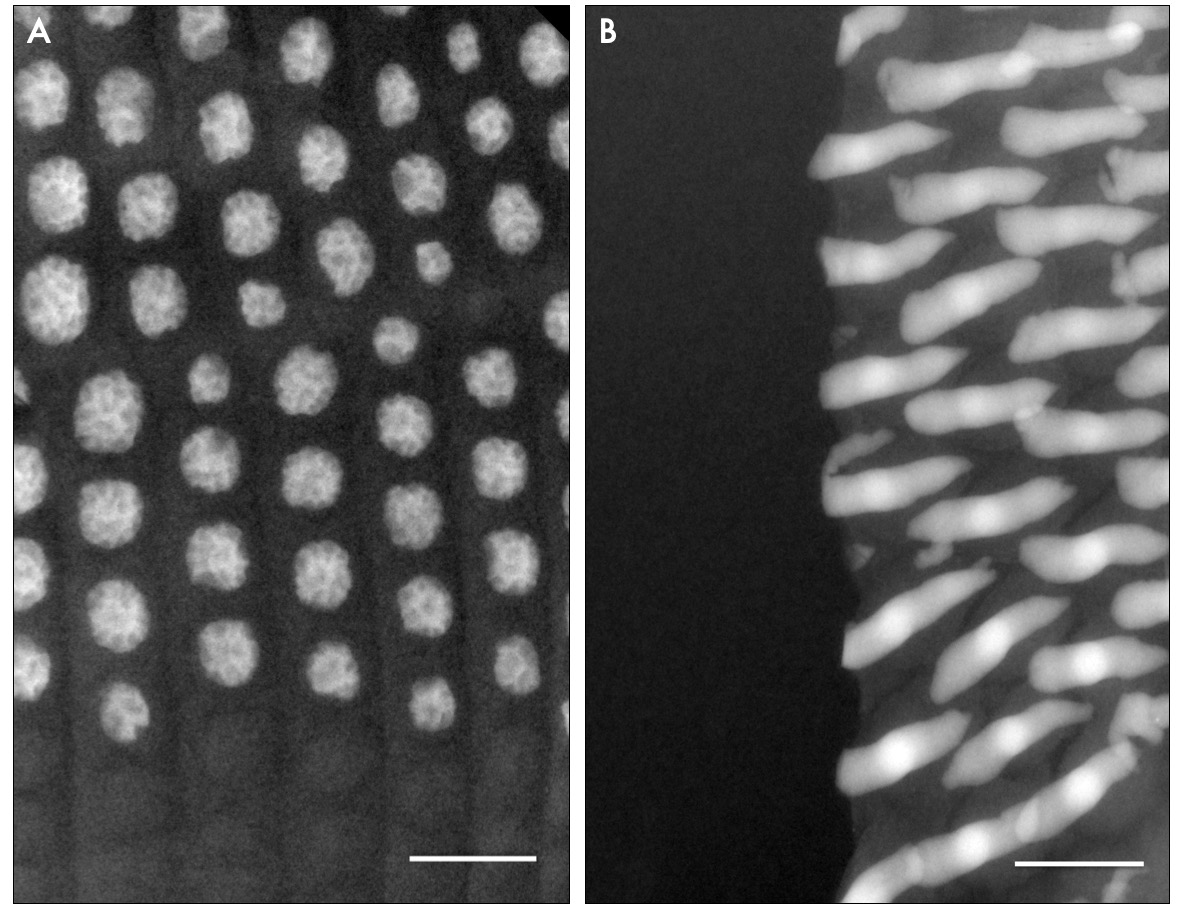


**Supplementary Figure 1:** X-ray plate imaging of the dorsal skin of (A) *Heloderma suspectum* and (B) *Varanus komodoensis.* White indicates a denser material, black indicates a less dense material. The mineralised osteoderms thus appear white in comparison to the non-mineralised surrounding tissue. There are substantial differences in the overall shape and degree of tessellation between the two species. Scale bars: 5mm


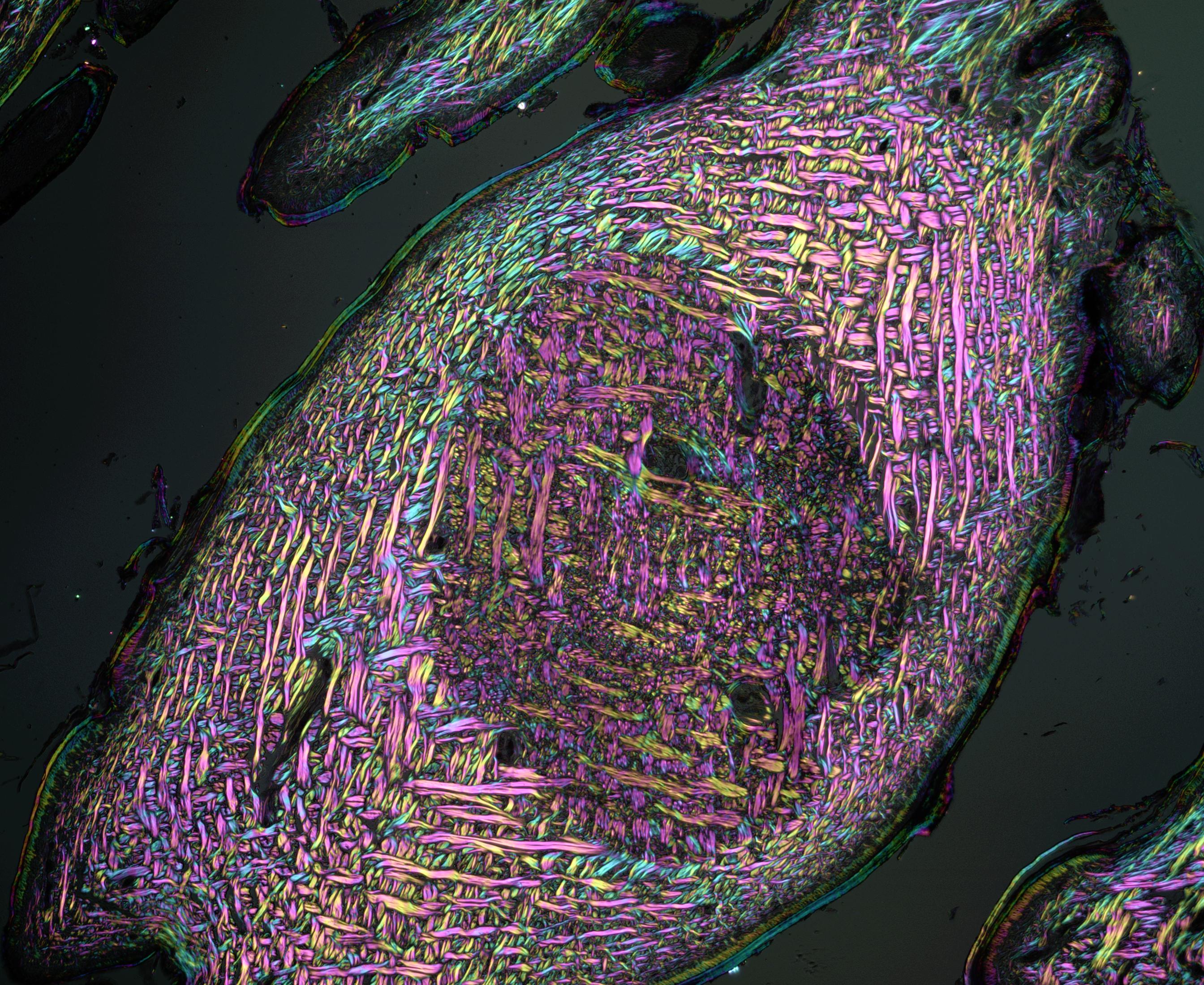


**Suggested Cover Image:** An uncropped version of the image shown in Figure 3C. Polarised light micrograph of a coronal section of *H. suspectum* osteoderm and surrounding dermis. False colour composite created from six images taken with crossed linearly polarising filters at 15º rotation intervals, merged in the colour circle sequence Red, Yellow, Green, Cyan, Blue, Magenta. Colour shows the orientation within the section plane, with four repeat cycles in 360º. Brightness is proportional to the cosine of the strike angle with respect to section plane, being brightest in plane, and black when perpendicular to that plane, i.e., parallel to the optic axis. Dark central patch corresponds to the region that was mineralised. Width of field = 3.04mm
